# Metabolomics identifies metabolite biomarkers associated with acute rejection after heart transplantation in rats

**DOI:** 10.1038/s41598-017-15761-3

**Published:** 2017-11-13

**Authors:** Feng Lin, Yi Ou, Chuan-Zhong Huang, Sheng-Zhe Lin, Yun-Bin Ye

**Affiliations:** 1 Department of Cardiovascular Surgery, Union Hospital, Fujian Medical University, Fuzhou, 350001 Fujian Province China; 2 Laboratory of Immuno-Oncology, Fujian Medical University Cancer Hospital, Fujian Provincial Key Laboratory of Translational Cancer Medicine, Fuzhou, 350014 Fujian Province China; 3 Union College of Clinical Medicine, Fujian Medical University, Fuzhou, 350122 Fujian Province China

## Abstract

The aim of this study was to identify metabolite biomarkers associated with acute rejection after heart transplantation in rats using a LC-MS-based metabolomics approach. A model of heterotopic cardiac xenotransplantation was established in rats, with Wistar rats as donors and SD rats as recipients. Blood and cardiac samples were collected from blank control rats (Group A), rats 5 (Group B) and 7 days (Group C) after heart transplantation, and pretreated rats 5 (Group D) and 7 days (Group E) post-transplantation for pathological and metabolomics analyses. We assessed International Society for Heart and Lung Transplantation (ISHLT) grades 0, 3B, 4, 1 and 1 rejection in groups A to E. There were 15 differential metabolites between groups A and B, 14 differential metabolites between groups A and C, and 10 differential metabolites between groups B and C. In addition, four common differential metabolites, including D-tagatose, choline, C16 sphinganine and D-glutamine, were identified between on days 5 and 7 post-transplantation. Our findings demonstrate that the panel of D-tagatose, choline, C16 sphinganine and D-glutamine exhibits a high sensitivity and specificity for the early diagnosis of acute rejection after heart transplantation, and LC-MS-based metabolomics approach has a potential value for screening post-transplantation biomarkers.

## Introduction

Heart transplantation has increasingly become the primary treatment for end-stage heart diseases^1–3^. Rejection is recognized as a major risk factor affecting the survival in patients undergoing heart transplantation^4–6^. Untimely diagnosis and treatment of rejection may cause irreversible damages to transplanted organs and even be life-threatening^5^. Immunosuppressant treatment has been proved to greatly reduce the incidence of acute rejection; however, acute rejection remains a major cause that affects the early survival of the transplanted organs post-transplantation^7–9^. Early diagnosis of acute rejection and the subsequent adjustment of immunosuppressive protocols is therefore of great importance to improve the survival in patients undergoing transplantation.

Currently, endomyocardial biopsy remains the “golden” standard for the diagnosis of post-transplantation rejection^10–12^; however, endomyocardial biopsy is an invasive procedure, which may cause a high incidence of complications and have a low compliance^13,14^. In addition, the transplanted organs often undergo irreversible damages upon on diagnosis of acute rejection by pathological examinations^15^. A search for non-invasive approaches with high sensitivity and specificity is urgently needed for the diagnosis of post-transplantation rejection.

Metabolomics, a quantitative measurement of the dynamic multiparametric metabolic response of living systems to pathophysiological stimuli or genetic modification^16^, has been widely used for the screening of biomarkers for disease diagnosis^17–20^. Metabolomics is able to reveal metabolites, which have the most direct associations with biological phenotypes^21^. The aim of this study was to identify metabolite biomarkers associated with acute rejection after heart transplantation in rats using non-targeted metabolomics, so as to provide potential candidates for the early diagnosis of post-transplantation acute rejection.

## Results

### Pathology of the myocardial tissues of the donor heart

**Figure 1:**
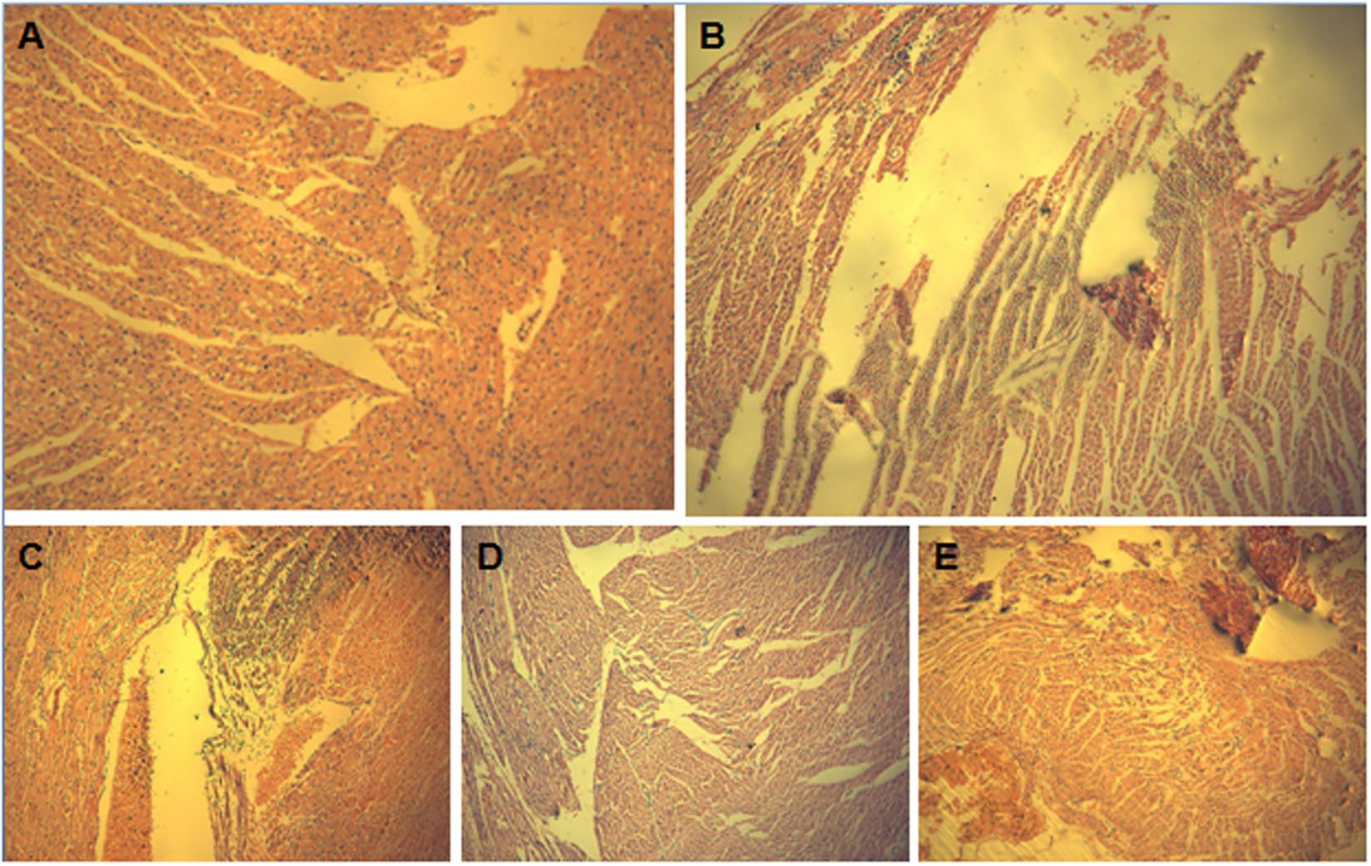
HE staining of rat myocardium (×100). (**A**) Normal myocardium; (**B**) myocardial tissues sampled 5 days after organ transplantation; (**C**) myocardial tissues sampled 7 days after organ transplantation; (**D**) rats are intraperitoneally injected with cyclosporine A at a dose of 20 mg/kg one day pre-transplantation and at a dose of 10 mg/kg post-transplantation for 5 days, and then myocardial tissues are sampled; (**E**) rats are intraperitoneally injected with cyclosporine A at a dose of 20 mg/kg one day pre-transplantation and at a dose of 10 mg/kg post-transplantation for 7 days, and then myocardial tissues are sampled.

International Society for Heart and Lung Transplantation (ISHLT) grade 0 rejection was assessed in the normal myocardium (Fig. 1A)^22^. In Group B, mild edema and necrosis of myocardial cells, and massive interstitial lymphocyte infiltration were seen, and ISHLT grade 3B rejection was assessed (Fig. 1B). In Group C, mild edema and necrosis of myocardial cells, massive interstitial lymphocyte and granulocyte infiltration, and a smaller amount of thrombus were observed, and ISHLT grade 4 rejection was assessed (Fig. 1C). In addition, no myocardial cell necrosis and a little massive interstitial lymphocyte infiltration were seen in groups D and E, with ISHLT grade 1 rejection assessed (Fig. 1D and E).

### Data quality assessment

**Figure 2:**
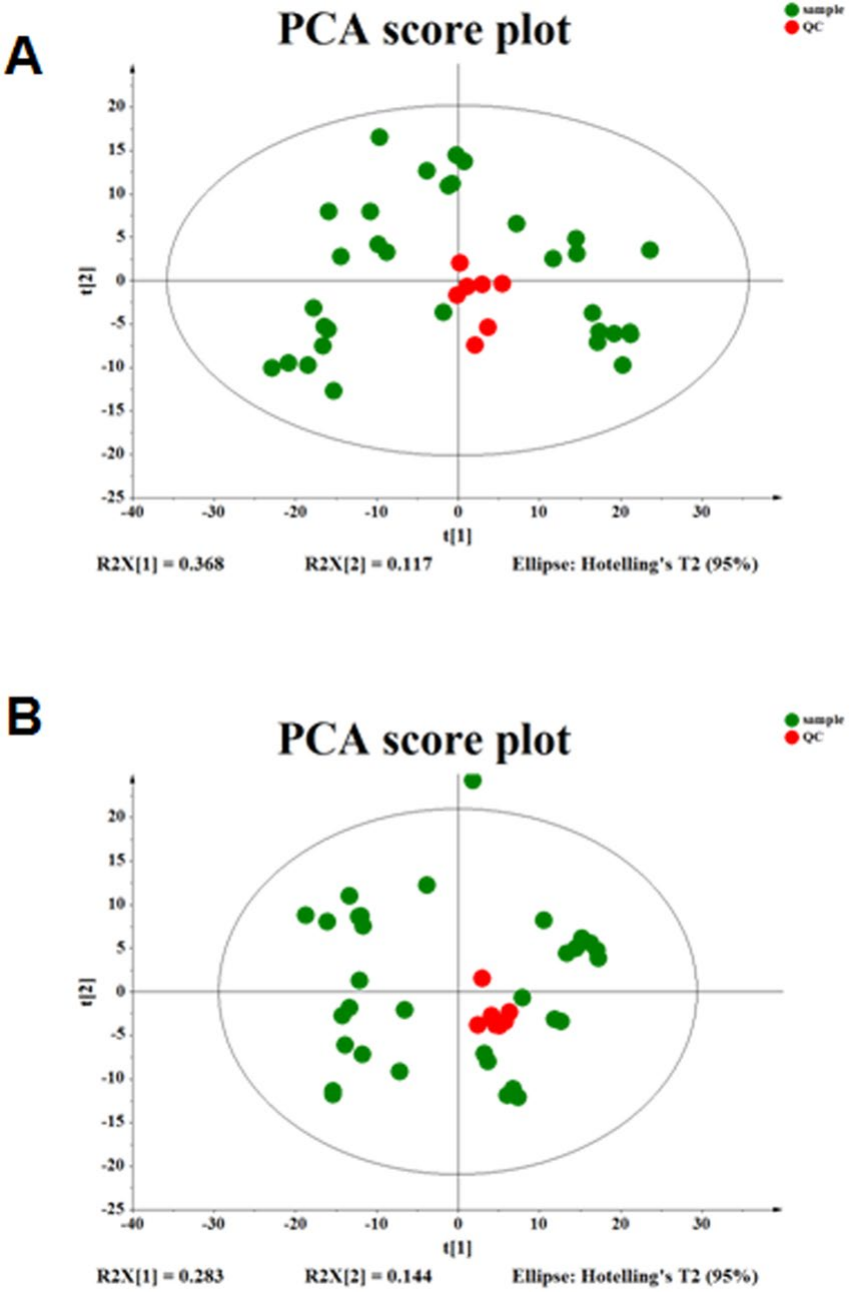
The principal component analysis (PCA) score plot of the quality control samples. All quality control (QC) samples are found to cluster in the PCA plot and the error in QC is within two standard deviations (SDs).

**Figure 3:**
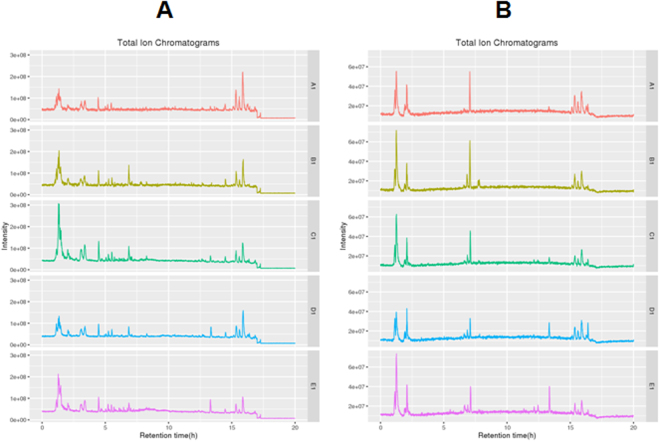
The typical total ion chromatograms (TICs) and base peak chromatograms (BPCs). (**A**) Positive ion; (**B**) negative ion.

Quality control (QC) and quality assurance (QA) are required in order to obtain reliable and high-quality data from liquid-chromatography-tandem-mass-spectrometry (LC-MS)-based metabolomics analysis, and the QA samples cluster is considered reliable if the error in QC is within two standard deviations (SDs). All QC samples were found to cluster in the principal component analysis (PCA) plot and within the 95% confidential intervals (CIs), and the error in QC was within two SDs (Fig. 2). Total ion chromatograms (TICs) and base peak chromatograms (BPCs) showed no drifting retention time or chromatographic shapes during the whole run-sequence (Fig. 3), indicating the LC-MS results are qualified for statistical analyses.

### Multivariate statistical analysis

**Figure 4:**
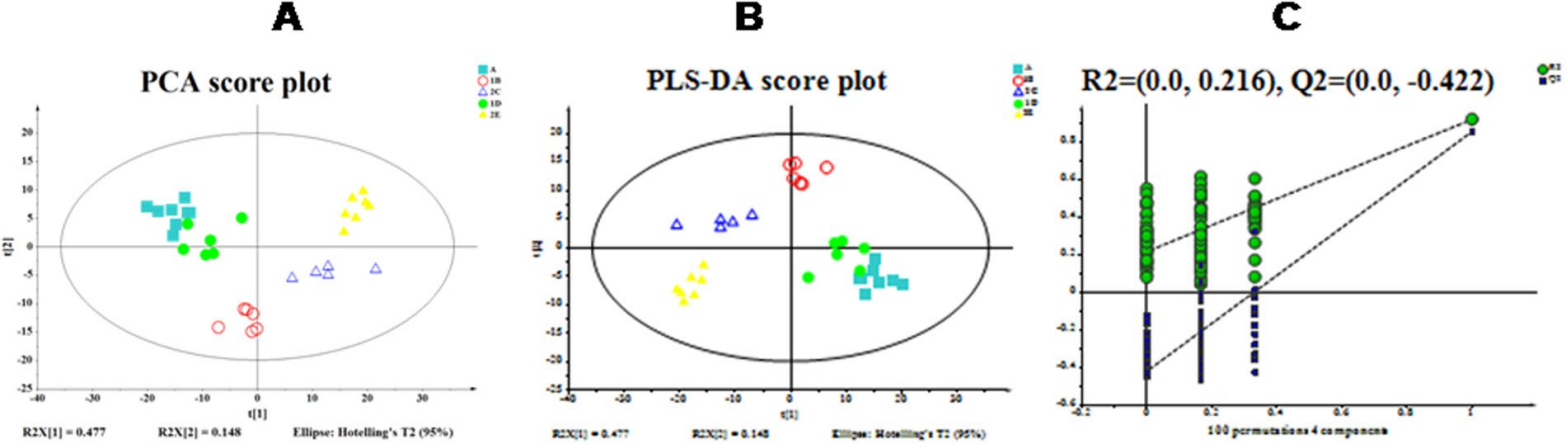
Principal component analysis (PCA) and partial least squares discriminant analysis (PLS-DA) score plots. (**A**) PCA score plot. The horizontal ordinate indicates the score of samples in the first principle component, and the vertical ordinate indicates the score of samples in the second principle component. R2X^1^, the interpretable degree of the first principle component; R2X^2^, the interpretable degree of the second principle component. (**B**) PLS-DA score plot; (**C**), permutation tests for the PLS-DA score plot. The horizontal ordinate indicates the score of samples in the first principle component, and the vertical ordinate indicates the score of samples in the second principle component. R2X^1^, the interpretable degree of the first principle component; R2X^2^, the interpretable degree of the second principle component.

Following pretreatment, the LC-MS data were subject to PCA and partial least square-discriminant analysis (PLS-DA), and permutation tests were performed to prevent the PLS-DA model overfitting^23,24^. In order to investigate the changes of the global metabolite profiles in the serum of rats given immunosuppressive therapy at acute rejection, the samples in the 5 groups were combined for PCA and PLS-DA. The results showed that the samples from the 5 groups were all distributed in the 95% CI PCA scores and appeared remarkable clusters and grouping (Fig. 4), indicating that immunosuppressive therapy exhibits a remarkable metabolic disturbance during acute rejection. The LC-MS data sets captured from the positive- and negative-ion modes were subject to the same grouping and statistical analyses, and significant clusters were found in the clustering of LC-MS spectral features among the groups A, B and C, and the groups A, B and D in the positive- and negative-ion modes, and among the groups A, C and E in the positive-ion mode. Taking the pathological examinations of the rat myocardial tissues, it is indicated that the metabolite profiles change during acute rejection.

### Identification and characterization of potential biomarkers

According to the PCA and PLS-DA results, we identified potential biomarkers based on a combination of an independent *t* test (*P* < 0.05), variable importance in project (VIP, ≥1), S-plot. pcorr and receiver operating characteristic (ROC) curve analysis of the differential metabolites. There were 15 differential metabolites identified between groups A and B, and ROC curve analysis revealed area under curve (AUC) values of >0.90 in 6 metabolites. We identified 14 differential metabolites between groups A and C, and 5 metabolites had AUC values of >0.90. In addition, there were 10 differential metabolites identified between groups B and C, and 2 metabolites had AUC values of >0.90 (Table 1).

**Table 1:** Metabolomics identifies differential metabolites between groups A and B, A and C, A and D, A and E, B and C, and C and E, and their areas under curve (AUCs).

Group	Metabolite	AUC
Group A vs. Group B	Choline	1.00
	C16 sphinganine	1.00
	D-glutauine	0.96
	Dysopc (15:0)	0.95
	l-pheuylalanine	0.93
	D-tagatose	0.91
Group A vs. Group C	Choline	1.00
	C16 sphinganine	1.00
	D-glutauine	0.98
	l-pheuylalanine	0.92
	Oxoglutaric acid	0.95
Group A vs. Group D	Choline	1.00
	C16 sphinganine	0.98
	D-glutauine	0.95
	Oxoglutaric acid	0.94
	D-tagatose	0.91
Group A vs. Group E	Choline	1.00
	C16 sphinganine	1.00
	D-tagatose	0.96
	Dysopc (15:0)	0.94
	D-glutauine	0.92
Group B vs. Group C	Oxoglutaric acid	0.94
	C16 sphinganine	0.98
Group C vs. Group E	Choline	1.00
	C16 sphinganine	1.00
	D-tagatose	0.94
	D-glutauine	0.92

On day 5 after heart transplantation, there were 14 differential metabolites identified between groups B and D, and ROC curve analysis revealed AUC values of >0.90 in 4 metabolites, including D-glutamine, choline, C16 sphinganine and D-tagatose (Table 2). We identified 13 differential metabolites between groups C and E 7 days after heart transplantation, and ROC curve analysis revealed AUC values of >0.90 in 5 metabolites, including D-tagatose, choline, lysopc (15:0), C16 sphinganine and D-glutamine. There were 15 differential metabolites between groups A and D, with AUC values of >0.90 in 5 metabolites, and there were 13 differential metabolites between groups A and E, with AUC values of >0.90 in 5 metabolites. In addition, there were 10 differential metabolites between groups D and E, without AUC values of >0.90 in any metabolites.

**Table 2:** Metabolite biomarkers associated with acute rejection after heart transplantation identified between Group B and Group D.

m/z	Retention time (min)	Metabolite	Group B (mean ± SD)	Group D (mean ± SD)	VIP	S-plot. pcorr	*P* value	AUC
147.0759	1.23	D-glutamine	3.84 ± 0.27	1.74 ± 0.19	2.98	−0.985	2.54E-08	0.92
409.2942	13.32	Muricholic acid	0.015 ± 0.007	0.27 ± 0.05	1.02	0.947	3.29E-07	0.83
431.2757	13.33	15-keto latanoprost	0.06 ± 0.02	0.52 ± 0.09	1.38	0.928	5.01E-07	0.86
373.2729	13.32	3-oxo-chol-11-enic acid	0.17 ± 0.06	2.64 ± 0,54	3.20	0.943	5.82E-07	0.78
522.3538	15.97	Mycinamicin VII	0.89 ± 0.06	1.32 ± 0.10	1.30	0.926	9.42E-06	0.72
343.2237	16.39	16,17-epoxy-DHA	0.19 ± 0.03	0.81 ± 0.18	1.57	0.949	1.23E-05	0.81
177.1019	4.15	Serotonin	0.47 ± 0.02	0.78 ± 0.09	1.11	0.902	1.42E-05	0.76
407.2786	13.37	Cholic acid	0.07 ± 0.03	1.42 ± 0.51	1.10	0.897	7.43E-05	0.87
179.0558	2.12	D-tagatose	1.15 ± 0.21	2.25 ± 0.10	1.41	0.952	2.73E-07	0.95
104.1066	1.23	Choline	3.01 ± 0.10	5.90 ± 1.23	3.30	0.873	0.00018	1
305.2468	16.72	Sideridiol	0.013 ± 0.003	0.46 ± 0.19	1.29	0.895	0.0002	0.76
246.1694	6.19	2-methylbutyroylcarnitine	0.61 ± 0.12	0.25 ± 0.10	1.14	−0.837	0.0003	0.87
114.0657	1.33	Creatinine	0.90 ± 0.17	0.55 ± 0.05	1.11	−0.871	0.0007	0.85
274.2734	12.66	C16 sphinganine	3.03 ± 0.75	5.41 ± 0.84	2.32	−0.93	0.013	0.97

m/z, mass-to-charge ratio; VIP, the importance of the variable in the PLS-DA; S-plot. pcorr, the reliability of the dataset X.

**Figure 5:**
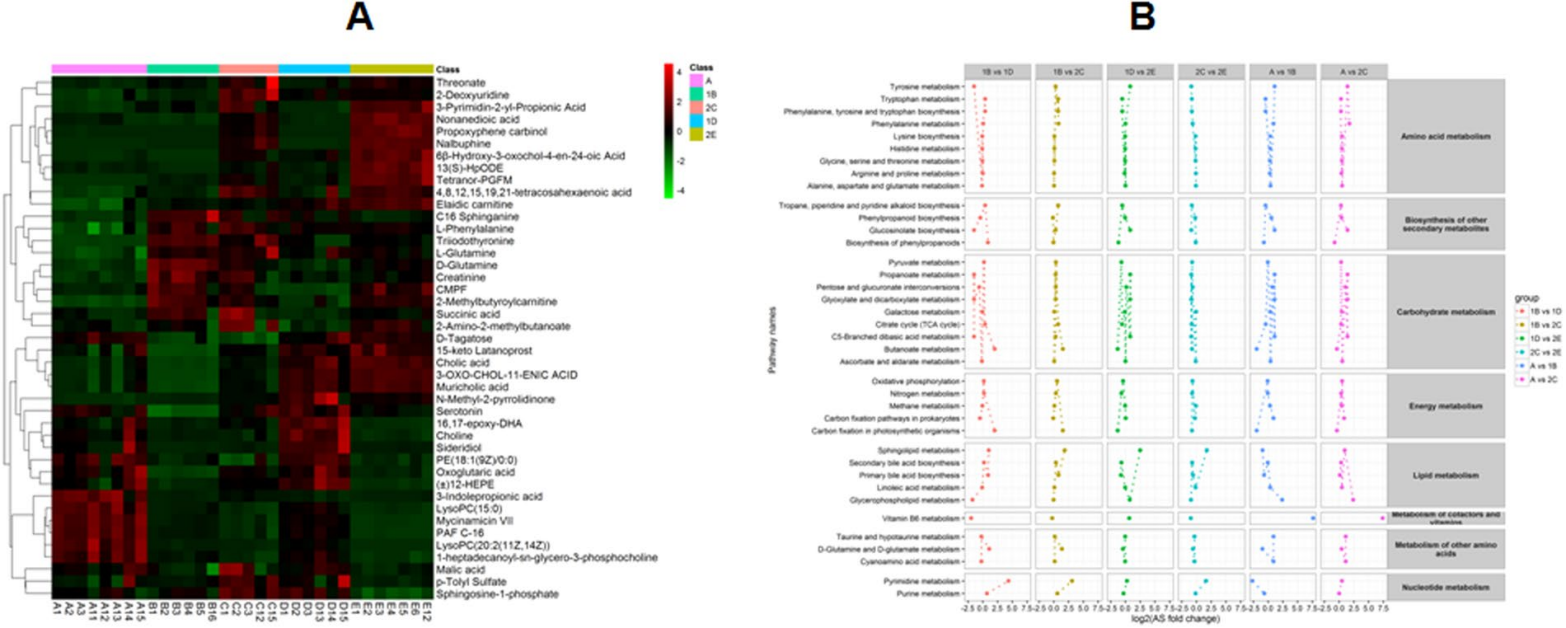
Hierarchical clustering and KEGG metabolic pathways of differential metabolites. (**A**) Heat maps of differential metabolites; (**B**) heat maps of KEGG metabolic pathways. Pathway activity profiling (PAPi) is used to calculate the activity score of each metabolic pathway. The heat maps are based on metabolic process and classified, and the scattergrams of the metabolic pathways are statistical analyses of the KEGG metabolic pathways (*P* < 0.05 as revealed by ANOVA).

Hierarchical clustering and KEGG metabolic pathways of the differential metabolites in the five groups are shown in Fig. 5. Low expression of threonate, 2-deoxyuridine and 3-pyrimindin-2-yl-propionic acid was seen in groups B and D 5 days post-transplantation, indicating no obvious alterations seen in these metabolites expression 5 days post-transplantation; however, the levels of these metabolites significantly increased 7 days post-transplantation. Moderate and low expression of C16 sphinganine, L-phenylalanine and triiodothyronine, and moderate and high expression of D-glutamine, 15-keto latanoprost and cholic acid was seen in groups D and E relative to in groups B and C. The data indicate that these metabolites are greatly affected by cyclosporine A, and may be indicative of the development of acute rejection. In addition, these metabolites are mainly involved in amino acid, fatty acid and bile acid metabolism, demonstrating that acute rejection may mainly affect substance metabolism pathways, but have few effects on energy metabolism pathways.

By comparing the differential metabolic biomarkers identified between on days 5 and 7 post-transplantation, there were four common differential metabolites, including D-tagatose, choline, C16 sphinganine and D-glutamine. During acute rejection, the serum D-tagatose level was significantly elevated, while the serum levels of choline, C16 sphinganine and D-glutamine were markedly decreased. These four potential metabolites were analyzed by logistic regression analysis and ROC curve analysis to build a panel of biomarkers. The results showed that the panel of the 4 potential metabolic biomarkers (D-tagatose, choline, C16 sphinganine and D-glutamine) had an AUC value of 0.983, and the sensitivity and specificity of the biomarker panel were 90.2% and 92.3%, respectively, at the best cut-off value. Our findings demonstrate that the panel of D-tagatose, choline, C16 sphinganine and D-glutamine may provide a higher value for the diagnosis of acute rejection after heart transplantation.

## Discussion

Acute rejection remains a major problem in heart transplantation until now, which may be life-threatening if timely therapy is not given^25^. However, there are no effective approaches for the early identification of acute rejection to date. A search for non-invasive, rapid and accurate early diagnosis of acute rejection after heart transplantation is therefore urgently needed. Proteomics has been widely used for screening transplantation-related biomarkers^26–30^; however, proteomics suffers from problems of acquisition of huge amounts of information and high detection costs^31^.

The term metabolomics was firstly introduced in 1999^32^. Until now, approximately 2500 metabolites have been identified in the human body^33^, which greatly reduces the screening difficulty and workload by using metabolomics. Based on the platforms such as LC-MS, nuclear magnetic resonance (NMR) spectroscopy ion-mobility spectrometry and electrochemical detection^34^, metabolomics is able to non-invasively, more accurately and more rapidly investigate drug metabolism and *in-vivo* metabolic changes in disease models with lower costs^35–38^.

Metabolomics approaches have been employed in organ transplantations^39–41^. NMR spectroscopy-based metabolomics analysis of the whole blood samples from a patient who underwent two consecutive liver transplantations showed distinctive metabolic changes 2 h after the first transplant surgery when no other variable or conventional laboratory tests indicated poor graft function, indicating the great value of metabolomics in the identification of transplantation-related biomarkers^42–44^. Currently, a huge lack of donors is a major crisis in organ transplantation; however, there are two key issues that remain unaddressed in organ transplantation, which are how to assess whether a donors after cardiac death (DCD) liver is damaged beyond repair, and whether machine perfusion has rendered an injured organ sufficiently viable for transplantation^45^. A metabolic analysis of the transient responses of cadaveric rat livers during normothermic machine perfusion revealed that four metabolites ornithine, arginine, albumin and tyrosine that varied significantly for ischemic livers enabled the evaluation of the organ ischemic injury level, and may be used to identify whether an organ is feasible for transplantation^46^. The metabolomic approach based on ultra performance liquid chromatography (UPLC) and quadrupole time-of-flight MS was considered as a feasible tool to investigate the metabolic abnormality in the acute graft rejection in renal transplantation^40^. In addition, metabolomics has shown promising values in the storage of donor kidneys^47^, cancers^48^ and heart diseases^49^.

In the current study, a rat model of heterotopic heart transplantation was established, and the post-transplantation heart can be considered as a tissue with ejection function alone. Therefore, the impact of liver metabolism on endogenous metabolites may be excluded relative to liver transplant models. Pathological examinations confirmed the successful modeling of heart transplantation on day 5 post-transplantation in Group B and on day 7 post-transplantation in Group C. Previous studies have shown that mild lymphocyte infiltration occurs in animal models of heart transplantation on day 2 post-transplantation, and peaks on day 8 post-transplantation. During this period, myocardial edema, degeneration and necrosis, and inflammatory cell infiltration are seen^50^. Ono and Lindsey also observed acute rejection 5 to 7 days post-transplantation in untreated rats^51^, which is consistent with the findings from the present study.

Currently, the mechanisms of acute rejection have not been completely demonstrated^52^. The primary mechanism for acute rejection is a T lymphocytes-mediated cellular immune response. Namely, antigen-activated T cells are aggregated in transplants and release active cytokines or cause a direct killing effect, resulting in immune injuries; in addition, ischemia-reperfusion injury is also a mechanism of acute rejection^52^. In this study, we investigated the metabolic profiles in the serum of rats undergoing heart transplantation using non-targeted metabolomics, and PCA, PLS-DA and ROC curve analysis was employed to compare the metabolic profiles between groups. The difference in metabolites levels between groups may indicate the apparent alterations of metabolites during acute rejection, and serum metabolomics may identify biomarkers for predicting and diagnosing acute rejection. In this study, there were 14 differential metabolites identified between groups B and D, including 5 types of amino acids (D-glutamine, muricholic acid, 3-oxo-chol-11-enic acid, cholic acid and creatinine), 3 types of lipids (15-keto latanoprost, mycinamicin VII and 16,17-epoxy-DHA), 2 types of alcohols (C16 sphinganine and sideridiol), 2 types of organic bases (choline and 2-methylbutyroylcarnitine), one saccharide (D-tagatose), and one indole derivative (serotonin). There were 11 metabolites down-regulated and 3 metabolites up-regulated in Group B, and these metabolites were found to be involved in amino acid (D-glutamine, 3-oxo-chol-11-enic acid, serotonin, choline, sideridiol and creatinine), fatty acid (16,17-epoxy-DHA, 2-methylbutyroylcarnitine and C16 sphinganine), bile acid (muricholic acid and cholic acid), drug (15-keto latanoprost and mycinamicin VII) and saccharide (D-tagatose) metabolism. The results demonstrate that cyclosporine A exhibits multiple effects on acute rejection in rats, and has the greatest impact on amino acid metabolism. As a polypeptide, cyclosporine A exhibits immunosuppressive functions through impacting T cell activity via the amino acid-protein interactions. Our data demonstrate that four metabolites, including choline, C16 sphinganine, D-tagatose and D-glutamine, may be used as biomarkers for the prediction and diagnosis of acute rejection after heart transplantation. Choline, which is involved in lipid metabolism, up-regulates the expression of HSP70 and Cox-2, two effectors of ischemic preconditioning, through activating M3 receptor, thereby alleviating myocardial ischemia-reperfusion injury^53^. In this study, choline was found to be significantly reduced in Group B than in Group D on day 5 post-transplantation, and in Group C than in Group E on day 7 post-transplantation. C16 sphinganine has been found active to protect ischemia/reperfusion-induced arrhythmias in the isolated heart, prevent allograft rejection during heart transplantation, promote cardiovascular regeneration and maintain myocardial cell survival during ischemia^54^. In addition, low C16 sphinganine was reported to promote the development of rejection^54^. We detected a significant reduction in serum C16 sphinganine in Group B than in Group D on day 5 post-transplantation, and in Group C than in Group E on day 7 post-transplantation. D-tagatose, which is involved in energy metabolism^55^, was found to significantly reduce in Group B than in Group D on day 5 post-transplantation, and in Group C than in Group E on day 7 post-transplantation. Metabolomics analysis revealed that glutamine, glycine and methionine were of great importance to induce immune tolerance after liver transplantation in rats, while the metabolites urocanic acid and etiocholanolone may affect the induction of immune tolerance^56^.

In this study, we identified two metabolic biomarkers, C16 sphinganine and oxoglutaric acid, by comparing the metabolic profiles between groups B and C. C16 sphinganine was found to significantly reduce on day 7 (Group C) in relative to day 5 (Group B) after heart transplantation, while oxoglutaric acid significantly increased. Oxoglutaric acid is involved in energy metabolism and tricarboxylic acid cycle to produce amino acid. As a precursor of glutamine, oxoglutaric acid may replace the function of glutamine in enteral nutrition and immune response, and it may enhance immune functions during liver transplantation^57^. It is hypothesized that elevation of oxoglutaric acid may aggravate acute rejection after heart transplantation through increasing immune functions. However, further studies are required to test the hypothesis. Pathological examinations showed aggravation of rejection in Group C than in Group B, suggesting that these two metabolites may be useful for identifying the degree of acute rejection.

KEGG pathway provides integration and interpretation of metabolic pathways, including metabolism of carbohydrates, nucleosides and amino acids and biodegradation of organic compounds, which may reveal the possible metabolic pathways^58^. In this study, KEGG pathway analysis revealed abnormal metabolism of amino acid, purine, pyrimidine, oxidative phosphorylation, and D-glutamine in rats during acute rejection.

The results of the present study demonstrate that the panel of D-tagatose, choline, C16 sphinganine and D-glutamine exhibits a high sensitivity and specificity for the early diagnosis of acute rejection after heart transplantation. The current study is the first to employ LC-MS to investigate the metabolomic alterations in the serum of rats undergoing heart transplantation, which provides basis for examining the role of related molecules in rejection following heart transplantation. The LC-MS-based metabolomics technique, a novel approach that may identify biomarkers associated with acute rejection after heart transplantation, has a potential value for screening post-transplantation biomarkers.

## Materials and Methods

### Ethical statement

This study was approved by the Ethical Review Committee of the Union Hospital of Fujian Medical University (approval number: FJXHYY2013-00127). All animal experimentations were performed according to the international and national guidelines for the management and care of laboratory animals.

### Animals

Forty donor male Wistar rats, each weighing 224.61 ± 12.10 g, and forty recipient male SD rats, each weighing 316.26 ± 11.61 g, were purchased from Wushi laboratory animal Co., Ltd. (Minhou, China). All animals were housed in a clean laboratory animal facility and given free access to food and water.

### Animal modeling and grouping

All Wistar and SD rats were randomly assigned into 5 groups, of 8 animals in each group. A rat model of heterotopic cardiac xenotransplantation was established using a modified Heron’s technique^59^, with Wistar rats as donors and SD rats as recipients. Blood and myocardial samples were collected from blank control rats (Group A). In two acute rejection groups, the recipient rats were not given any pretreatment, and blood and myocardial samples were collected 5 (Group B) and 7 days (Group C) after heart transplantation. In two pretreatment groups, the recipient rats were intraperitoneally injected with cyclosporine A (Novartis Pharmaceuticals Corporation; East Hanover, NJ, China) at a dose of 20 mg/kg one day prior to transplantation and at a daily dose of 10 mg/kg post-transplantation. Briefly, cyclosporine A (250 mg/5 ml) was added into 95 ml normal saline to produce a solution at 2.5 mg/ml. For a rat weighing 300 g, 2.4 ml of cyclosporine A solution was given by intraperitoneal injection prior to transplantation and 1.2 ml was given post-transplantation. Blood and cardiac samples were collected 5 (Group D) and 7 days (Group E) post-transplantation.

### Blood collection

Rats were anesthetized with intraperitoneal injection of 3% pentobarbital sodium (Maixin Biological Technology Development Co., Ltd.; Fuzhou, China) at a single dose of 45 mg/kg. Then, rats were sterilized, and the abdominal wall was incised along the median line of the abdomen. The inferior cava vena was exposed, and 3 ml of blood samples were collected by puncture of the inferior vena cava. The collected blood samples were slowly transferred to additive-free blood collection tubes, stored at 4 °C for 2 h, and centrifuged at 4 °C, 3000 r/min for 10 min. The supernatant was carefully collected, and stored at −80 °C for the subsequent experiments.

### Pathological examinations

Following blood collection, the skin over the neck was incised along the original incision in rats receiving heart transplantation, and the transplanted heart was rapidly dissociated and extracted. In control rats, an incision was made on the median chest, and the heart was rapidly dissociated and extracted. Cardiac specimens were fixed in 10% neutral buffered formalin for more than 24 h, embedded in paraffin, cut into 5 µm sections, and subject to hematoxylin & eosin (HE) staining. The rejection was graded according to the criteria proposed by the International Society for Heart and Lung Transplantation^22^.

### Metabolomics analysis

All samples were thawed at 4 °C, and 100 µL of each sample was transferred to 1.5 ml centrifuge tubes. Then, each tube was added with 200 µL of methanol (pre-cooled at −20 °C) and vortexed for 60 s. The samples were centrifuged at 4 °C, 12000 r/min for 10 min, and the supernatant was collected into another 1.5 mL centrifuge tubes. The samples were then filtered through a 0.22 µm membrane, and the extracts were collected for LC-MS analysis, while 20 µL from each extract and mixture served as QC samples. These QC samples were used to monitor deviations of the analytical results from these pool mixtures and compare them to the errors caused by the analytical instrument itself.

Chromatographic separation was accomplished in an Acquity UPLC system (Waters Corporation; Milford, MA, USA) equipped with an ACQUITY UPLC® HSS T3 (150 × 2.1 mm, 1.8 µm) column maintained at 35 °C. The temperature of the autosampler was 8 °C. Gradient elution of analytes was carried out with 0.1% formic acid in water (A) and 0.1% formic acid in acetonitrile (B) at a flow rate of 0.3 ml/min. Injection of 3 μL of each sample was done after equilibration. An increasing linear gradient of solvent B (*v*/*v*) was used as follows: 0 to 1 min, 2% B; 1 to 11 min, 2% to 50% B; 11 to 17 min, 50% to 98% B; 17 to 18 min, 98% B; 18 to 19 min, 98% to 2% B; 19 to 20 min, 2%.

The ESI-MSn experiments were executed on the Thermo LTQ-Orbitrap XL mass spectrometer (Thermo Fisher Scientific, Inc.; Waltham, MA, USA) with the spray voltage of 4.8 kV and −4.5 kV in positive and negative modes, respectively. Sheath gas and auxiliary gas were set at 45 and 10 arbitrary units, respectively. The capillary temperature was 325 °C. The voltages of capillary and tube were 35 V and 50 V, −15 V and −50 V in positive and negative modes, respectively. The Orbitrap analyzer scanned over a mass range of m/z 50 to 1000 for full scan at a mass resolution of 60000. Data dependent acquisition (DDA) MS/MS experiments were performed with CID scan. The normalized collision energy was 30 eV. Dynamic exclusion was implemented with a repeat count of 2, and exclusion duration of 15 s.

### Data management and multivariate statistical analysis

The original LC-MS data were transformed into mzXML format using the software ProteoWizard version 3.0.8789 ^60^. Peaks identification, peaks filtration and peaks alignment were done using the XCMS program in R package version 3.1.3, to obtain data matrix containing mass-to-charge ratio (m/z), retention time and peak intensity. Then, the data matrix was subject to the following multivariate statistical analyses, including PCA and PLS-DA, and permutation tests were conducted to avoid the PLS-DA model overfitting.

The criteria for a differential metabolite included a *P* value of < 0.05 as revealed by an independent *t* test, VIP ≥ 1, and S-plot. pcorr ≥ 0.8 ^61^, a variable with VIP ≥ 1 indicated that the importance of this variable in the project was higher than the mean importance^62^.

### Metabolite identification and characterization

All metabolites were validated and annotated in the Human Metabolome Database (HMDS, http://www.hmdb.ca/), METLIN metabolite database (http://metlin.scripps.edu/landing_page.php?pgcontent=mainPage) and the standard sample database built by Yancheng Zhijian Biotechnology Cooperation (Yancheng, China), and the identified differential metabolites were evaluated for potential biomarkers using ROC curve analysis. An AUC value of >0.9 indicated a high accuracy for the prediction.
